# Intraventricular IL-17A administration activates microglia and alters their localization in the mouse embryo cerebral cortex

**DOI:** 10.1186/s13041-020-00635-z

**Published:** 2020-06-16

**Authors:** Tetsuya Sasaki, Saki Tome, Yosuke Takei

**Affiliations:** 1 Department of Anatomy and Neuroscience, Faculty of Medicine, University of Tsukuba, 1-1-1 Tennodai, Tsukuba, Ibaraki, 305-8577 Japan; 2 PhD Program of Neurosciences, Degree Program of Comprehensive Human Sciences, Graduate School of Comprehensive Human Sciences, University of Tsukuba, 1-1-1 Tennodai, Tsukuba, Ibaraki, 305-8577 Japan

**Keywords:** Autism Spectrum disorder, CD68, Corticogenesis, IL-17A, Microglia

## Abstract

Viral infection during pregnancy has been suggested to increase the probability of autism spectrum disorder (ASD) in offspring via the phenomenon of maternal immune activation (MIA). This has been modeled in rodents. Maternal T helper 17 cells and the effector cytokine, interleukin 17A (IL-17A), play a central role in MIA-induced behavioral abnormalities and cortical dysgenesis, termed cortical patch. However, it is unclear how IL-17A acts on fetal brain cells to cause ASD pathologies. To assess the effect of IL-17A on cortical development, we directly administered IL-17A into the lateral ventricles of the fetal mouse brain. We analyzed injected brains focusing on microglia, which express IL-17A receptors. We found that IL-17A activated microglia and altered their localization in the cerebral cortex. Our data indicate that IL-17A activates cortical microglia, which leads to a cascade of ASD-related brain pathologies, including excessive phagocytosis of neural progenitor cells in the ventricular zone.

## Introduction

Maternal immune activation (MIA) has been suggested to affect fetal brain development and to cause autism spectrum disorder (ASD) symptoms. Interleukin 17A (IL-17A) is a candidate mediator responsible for MIA-induced ASD pathogenesis [1–4]. In a commonly used mouse model of MIA, polyinosinic-polycytidylic acid [poly(I:C)], a viral mimic that potently induces inflammation, is administered to pregnant female mice [5, 6]. Offspring of poly(I:C)-treated mice exhibit behavioral phenotypes reminiscent of ASD, such as impaired social behavior and increased stereotypy, and ‘patch-like’ disorganized cytoarchitecture of the cerebral cortex [7]. Injection of recombinant IL-17A into fetal brain ventricles results in similar behavioral and histopathological abnormalities [1]. These animal models that reproduce cortical abnormalities are useful for understanding the pathogenesis of ASD. However, it has remained unclear how IL-17A acts on fetal brain cells and leads to ASD-like pathology.

Microglia are immune cells in the central nervous system similar to macrophages that phagocytose dead cells and debris [8, 9]. Microglia are derived from progenitor cells present in the yolk sac, and colonize the cerebral cortex from 4 to 24 weeks of gestation in humans and from about embryonic day (E)10 in mice [10]. In the resting state, microglia have small cell bodies and extend multiple protrusions (ramified-type microglia). However, when an inflammatory event occurs, their morphology becomes amoeboid-like with large cell bodies (amoeboid-type microglia) and they become highly phagocytic and release cytokines to cause cell damage [11]. In neural development, microglia are thought to play important roles in neuronal differentiation, maturation and regulation of neuronal death, and to contribute to neural circuit formation by phagocytosing unnecessary synapses and by contacting synapses [12–15].

In ASD, levels of inflammatory cytokines (e.g., TNFα, IL-6, and IFNγ) and chemokines (IL-8) are increased in the brain, indicating that the brain is in a weak inflammatory state [16]. It has also been suggested that microglial activation is involved in MIA-related behavioral abnormalities. For example, MIA alters the transcriptional profile of phagocytosis-related and cell motility-related genes in microglia [17]. Furthermore, behavioral abnormalities in MIA mice were improved by suppressing microglial activation by administration of minocycline and luteolin [17]. These findings indicate that MIA alters the microglial activity profile in the fetal mouse brain and may cause neuronal abnormalities via microglia and, thus, chronic neuroinflammation.

Microglia also express IL-17RA, a subunit of the IL-17A receptor [18]. In mouse models of multiple sclerosis and Parkinson’s disease [19], IL-17A induces overexpression of proinflammatory mediators of microglia and exacerbate neuroinflammation. In vitro studies also show that IL-17A stimulation increases the microglial expression of inflammatory chemokines and cytokines, including IL-6 and CXCL2 [20]. However, while these studies suggest relationships between IL-17A and microglia, the data were obtained using adult mice or in vitro cell cultures. It is not currently known how IL-17A affects microglia during neural development in vivo.

In this study, we examined the effect of IL-17A, focusing on possible alterations in the number and characteristics of microglia. We administered recombinant IL-17A into fetal mouse ventricles at E14.5, which is the stage of neuronal cell production. We show that localization of Cluster of Differentiation 68 (CD68)^+^ phagocytic microglia was biased medially in the cerebral cortex and mainly localized around the ventricular surface after IL-17A administration.

## Materials and methods

### Animals

C57BL/6 J mice were obtained from Japan CLEA (Tokyo, Japan). All animals were housed under standard laboratory conditions (12/12 h light/dark cycle, with free access to food and water). One or two female mice were mated in a cage containing one male. On the morning of the next day, the vaginal plug was confirmed. E0.5 was set at 12:00 on this day.

### Intraventricular administration of recombinant IL-17A to E14.5 embryos

Pregnant female mice at 14.5 days gestation were anesthetized with isoflurane. The uterus was carefully removed by caudal ventral midline incision and each fetus was identified. Recombinant IL-17A (0.6 μg/ml, R&D Systems, Minneapolis, MN) in Fast Green/saline (0.3 mg/ml) was administered into fetal ventricles using Fisherbrand™ Microhematocrit Capillary Tubes (Fisher Scientific, Hampton, NH). The control group received 2 μl of the Fast Green/saline (0.3 mg/ml). The success of all fetal intraventricular injections was assessed by staining the lateral ventricle with Fast Green. After administration, the uterus was returned to the abdominal cavity, the peritoneum was sutured with nylon thread, and the skin was sutured with silk thread, and the pregnant mice were recovered on a hot plate at 43 °C.

### Immunohistochemistry

E14.5 saline/IL-17A intracerebroventricularly-injected mice were removed from the uterus at E18.5. For histological analysis, brains were fixed overnight in 4% paraformaldehyde (PFA)/0.1 M phosphate buffer (PB) at 4 °C, and then immersed in 30% sucrose in 0.1 M PB at 4 °C until the tissue sank. Brain sections (40 μm) were prepared with a sliding microtome (REM-710, Yamato-Kohki, Japan) and stored at − 20 °C in cryoprotectant solution (30% glycerol, 30% ethylene glycol, 40% 0.1 M PB) until use.

The sections were rinsed with phosphate-buffered saline (PBS) three times, incubated in blocking solution (1% bovine serum albumin, 0.3% Triton-X 100, 0.1% NaN_3_ in PBS) for 60 min at room temperature, and then with anti-Iba1 (1:500, 019–19,741, FUJIFILM-Wako, Osaka, Japan) and anti-CD68 [1:200, MCA1957 (FA-11), BIO-RAD, Hercules, CA] antibodies for overnight at 4 °C. After washing three times with PBS, sections were incubated with F (ab’)2-Goat anti-Rabbit IgG (H + L) secondary antibody, Alexa Fluor 488 (1:500, A11077, Invitrogen, Carlsbad, CA) and Goat anti-Rat IgG (H + L) secondary antibody, Alexa Fluor 568 (1:500, A21069, Invitrogen) in blocking solution for 3 h at room temperature. Sections were then washed three times with in PBS containing 0.05% Tween-20, treated with 4′,6-diamidino-2-phenylindole (DAPI; 1:1000, Thermo Fisher Scientific, Waltham, MA) in PBS to visualize nuclei, washed three times with PBS, and mounted on glass slides and coverslipped (Matsunami Glass, Japan) using PermaFluor Aqueous Mounting Medium (Thermo Fisher Scientific).

### Image analysis

**Fig. 1:**
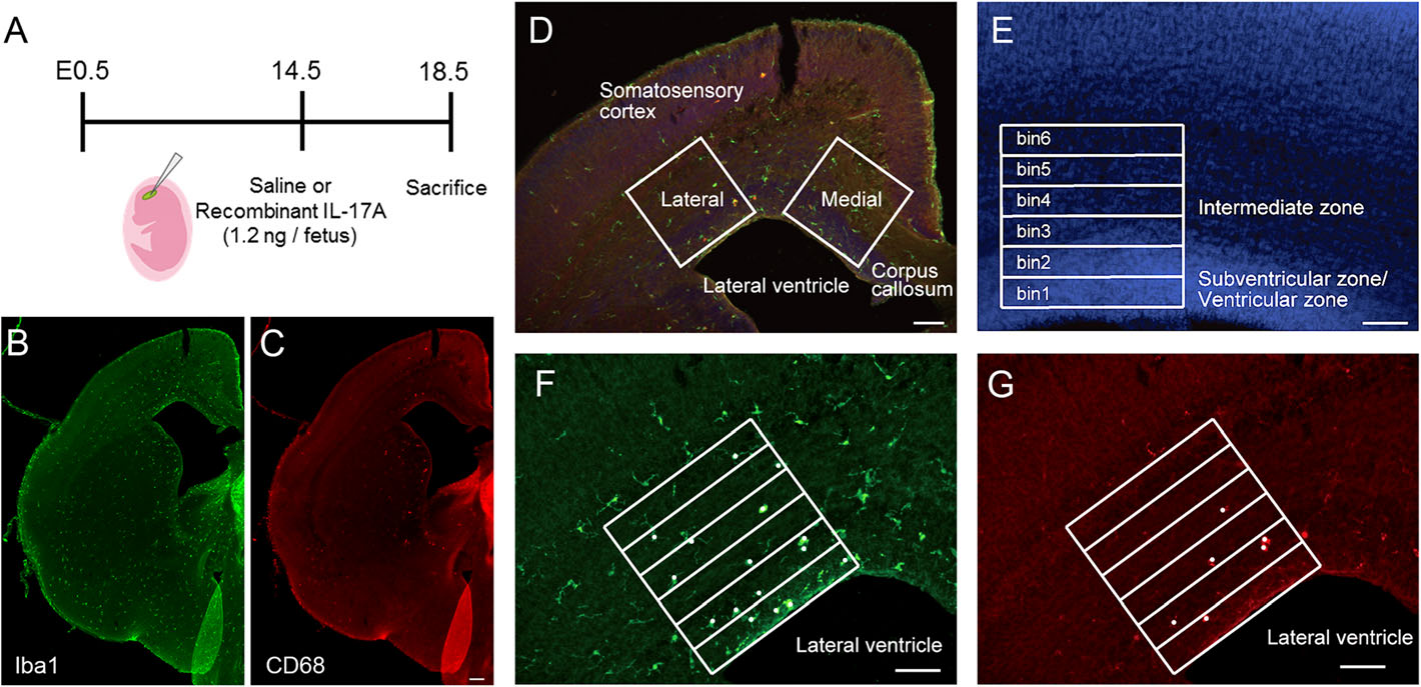
Experimental design and measurement of Iba1^+^ cells and CD68^+^ microglia in E18.5 cortex. **a** Experimental design of IL-17A injection to the lateral ventricle and brain sampling. Immunostaining of Iba1 (**b**) and CD68 (**c**) in E18.5 mouse brain. **d** Examples of ROIs (300 × 300 μm) placed on cingulate (medial) and primary sensory (lateral) cortex. **b** Position of bins 1 to 6 (each 50 × 300 μm) in the ROI. Example of counting Iba1 cells (**c**) and CD68 microglia (**d**). In all images, left is lateral and right is the medial side of the brain. Green: Iba1, Red: CD68, Blue: DAPI, Scale bars = 100 μm. CC: corpus callosum, LV: lateral ventricle, SC: somatosensory cortex, SVZ: subventricular zone, VZ: ventricular zone

**Fig. 2:**
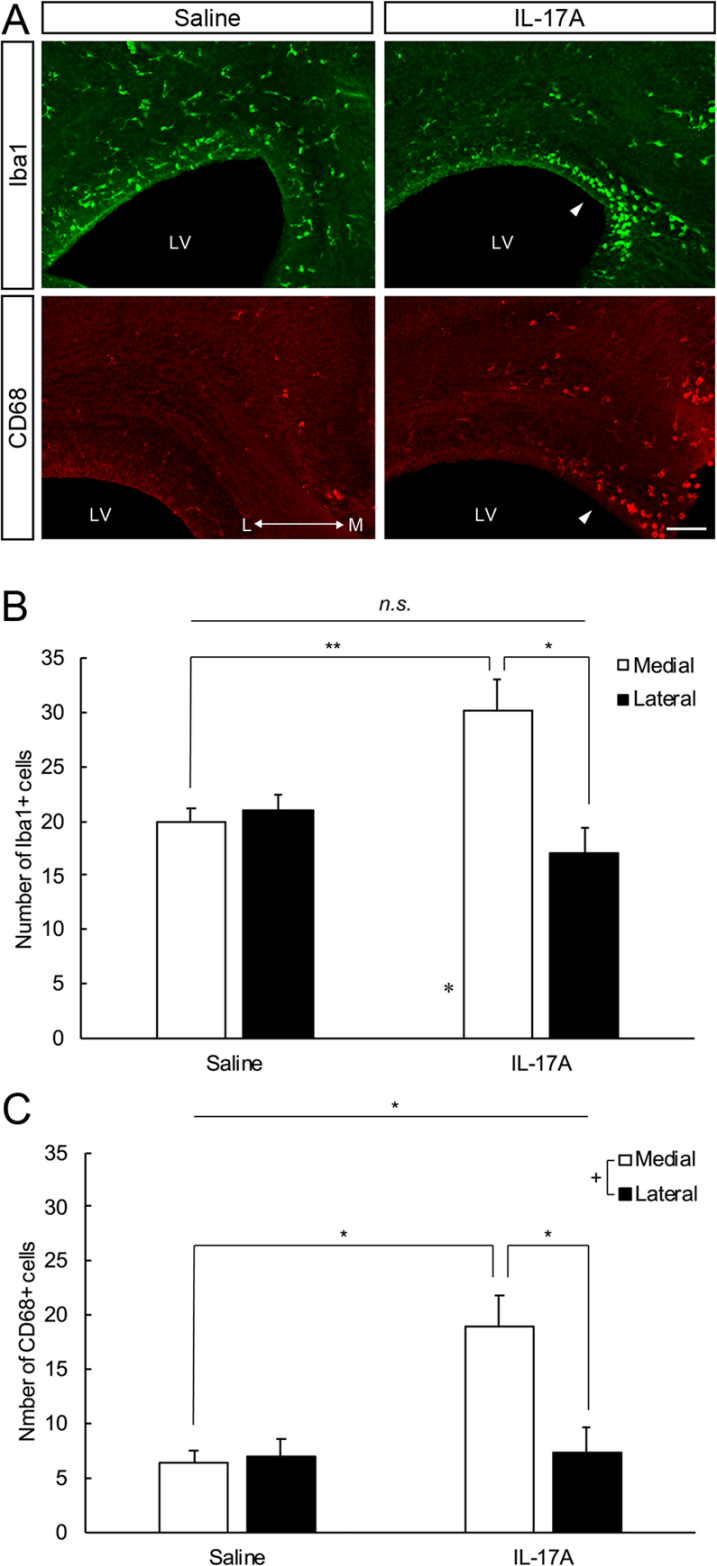
**a** Representative photographs of Iba1 (upper panel) and CD68 immunostaining (lower panel) after exposure to saline (left) and IL-17A (right). Arrowheads indicate accumulated microglia. LV: lateral ventricle. Scale bar = 100 μm. **b** Effects of IL-17A administration on microglia. Iba1 microglia in cingulate (medial) and somatosensory (lateral) cortices. IL-17A administration at E14.5 did not change the total number of microglia, but increased their tendency to accumulate in the medial region of the cortex. *N* = 6 per group, two-way ANOVA, * *p* < 0.05. **c** Effects of IL-17A administration on activated microglia. CD68 microglia in cingulate (medial) and somatosensory (lateral) cortices. IL-17A administration at E14.5 increased the number of amoeboid-type microglia especially in the medial region of the cortex. *N* = 6 per group, two-way ANOVA, * *p* < 0.05

**Fig. 3:**
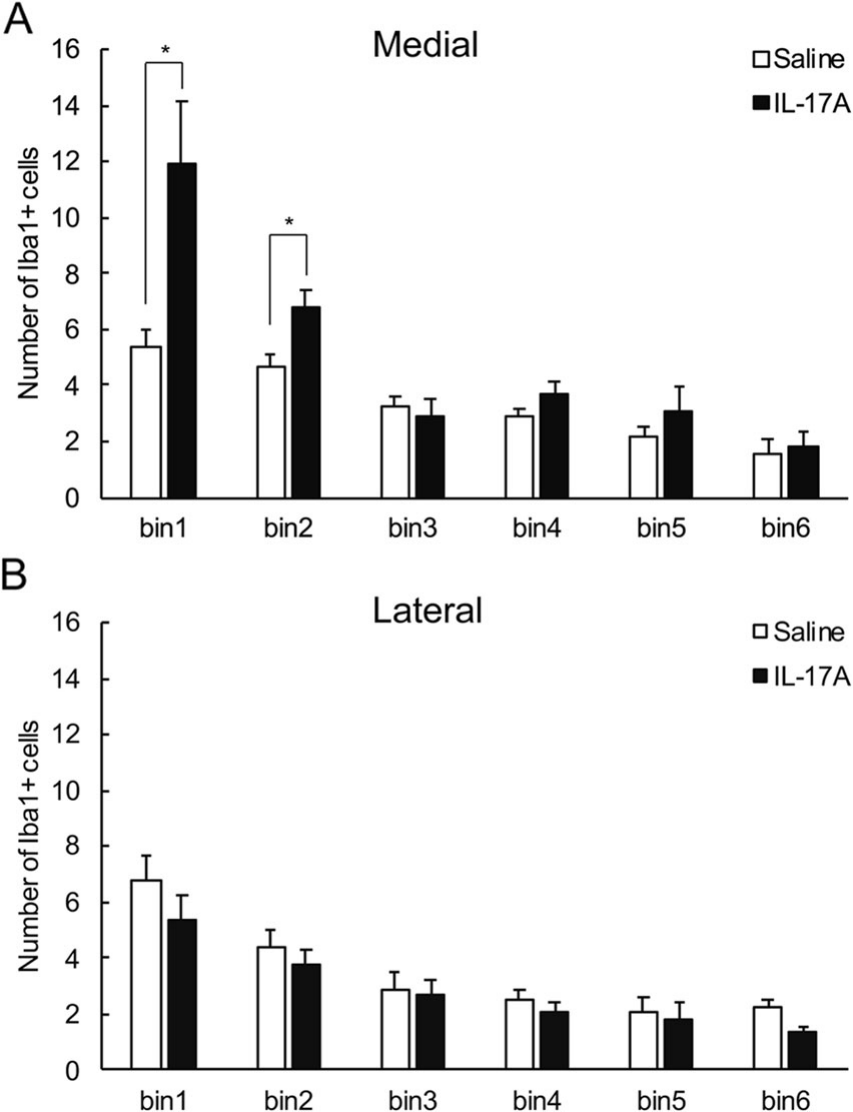
Bin-wise distribution of Iba1^+^ microglia in cingulate (medial, **a**) and somatosensory (lateral, **b**) cortices. IL-17A administration causes microglial accumulation in ventricular and subventricular zones in the medial region of the cortex. *N* = 6 per group, two-way ANOVA, * *p* < 0.05

**Fig. 4:**
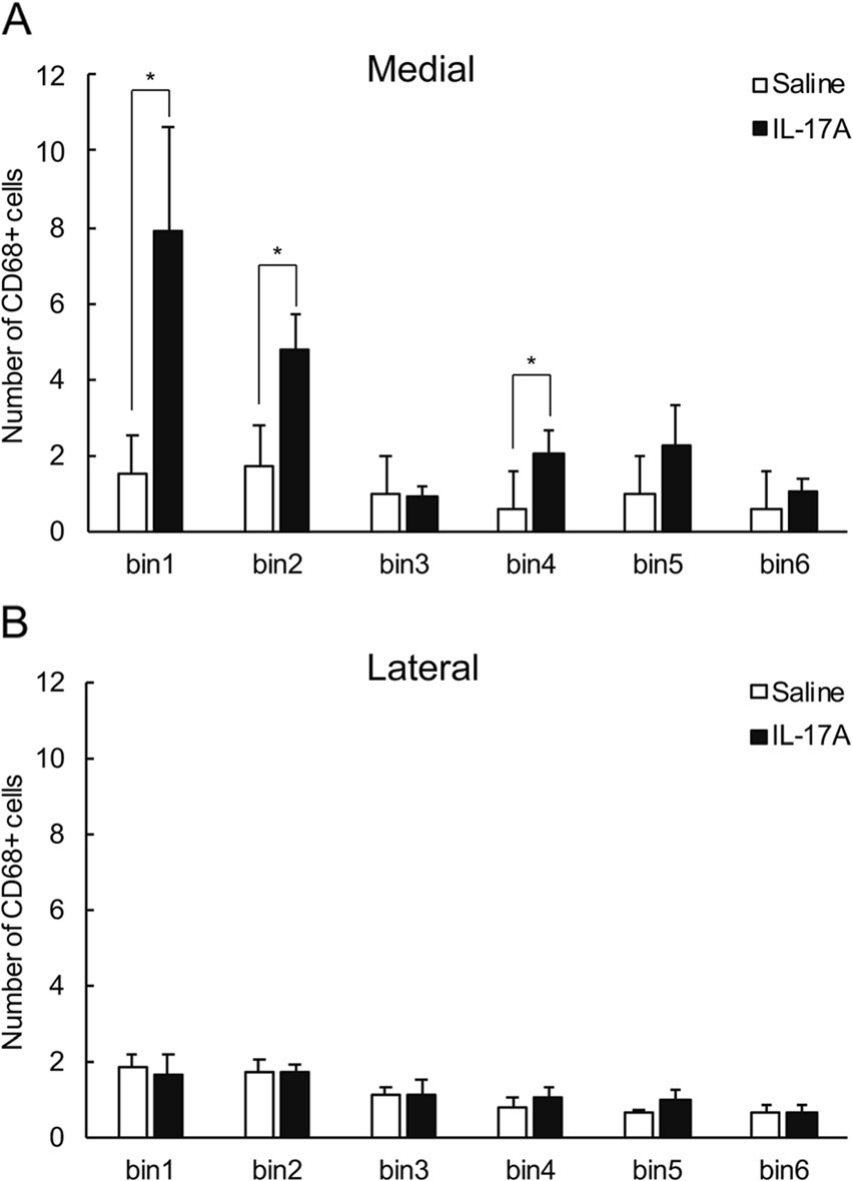
Bin-wise distribution of CD68^+^ microglia in cingulate (medial, **a**) and somatosensory (lateral, **b**) cortices. IL-17A administration causes accumulation of CD68^+^-activated microglia in ventricular and subventricular zones in the medial part of the cortex. *N* = 6 per group, two-way ANOVA, * *p* < 0.05

Fluorescence images were acquired using an All-in-One Fluorescence Microscope BZX-710 (Keyence, Japan). Two regions of interest (ROIs) of 300 × 300 μm were placed on the cortex (the cingulate cortex as a medial region and the somatosensory cortex as a lateral region) and were divided into six bins of 50 μm from the ventricle side (Fig. 1). The numbers of Iba1^+^ cells and CD68 cells in each ROI (Fig. 2) or bin (Figs. 3 and 4) were counted.

### Statistical analysis

Two-way analysis of variance (ANOVA) and Shaffer’s modified sequentially rejective Bonferroni post-hoc test was used. All statistical analyses were performed using R software. Probability values < 0.1 were considered marginally significant and probability values < 0.05 were considered significant. All data are expressed as the mean ± standard error of the mean.

## Results

IL-17A binds to a receptor consisting of IL17RA complexed with IL17RC, which activates downstream pathways including NFκB, MAPK, and C/EBP, and increases the expression of anti-microbial peptides, cytokines, and chemokines in target cells to induce an inflammatory response [21]. IL-17A has an activity of inducing expression of granulocyte-colony stimulating factor (G-CSF) and CXCL8 (IL-8), which causes activation of neutrophils and their migration to inflammatory sites [22]. In the brain, IL-17RA is expressed in microglia [18, 23]. To examine the effect of IL-17A on microglia in the mid-fetal period, we examined the brains of E18.5 mouse embryos treated with saline (Saline group) or IL-17A (IL-17A group) via the ventricle at E14.5 (Fig. 1). Immunostaining was performed using an antibody against ionized calcium binding adaptor molecule 1 (Iba1), a microglial/macrophage marker.

The number of Iba1^+^ microglia was not different between the groups [Fig. 2a, b, two-way ANOVA; effect of drugs: F (1, 10) = 2.88, N.S.], whereas interaction between groups and position was observed [two-way ANOVA; drug × location interaction: F (1, 10) = 9.92, *p* < 0.05]. In the IL-17A administration group, more Iba1^+^ microglia were accumulated in the medial region (cingulate cortex) than in the lateral region (somatosensory cortex) at E18.5.

A greater number of microglia had round morphology in mice treated with IL-17A compared with the saline group (Supplemental Fig. 1). This observation led us to examine the expression of CD68, a lysosomal marker specific to microglia that increases during phagocytosis [24, 25] to confirm the change in microglial properties caused by IL-17A administration. There was a difference between groups in the number of CD68^+^ cells [Fig. 2a, c, two-way ANOVA; effect of drugs: F (1, 10) = 5.57, *p* < 0.05] and in the interaction between groups and location [two-way ANOVA; drug × location interaction: F (1, 10) = 5.42, *p* < 0.05]. Thus, IL-17A administration at E14.5 increased the number of CD68^+^ microglia. In particular, microglia with increased phagocytic activity were accumulated in the medial region of cortices.

A ramified form of microglia was frequently observed in the gray matter, whereas round type microglia was observed on the ventricular surface in the E18.5 mouse brain (Supplemental Figs. 2 and 3). To evaluate the microglial distribution in the perpendicular direction (pia to ventricle axis), the number of cells in a bin was counted by dividing the ROI into bins every 50 μm (Fig. 1e, f and g). Iba1^+^ microglia in the medial region showed a difference between groups [Fig. 3a, two-way ANOVA; effect of drug: F (1, 10) = 11.26, *p* < 0.01], and interaction with group and bin was observed [drug × bin: F (5, 50) = 4.73, *p* < 0.01], which indicated that the number of Iba1^+^ microglia on the ventricular side (bins 1 and 2) was higher in the IL-17A-treated group. However, there was no difference between groups in the distribution of Iba1^+^ cell numbers in the lateral region [Fig. 3b, effect of drugs: F (1, 50) = 0.19, N.S.]. For CD68 microglia, an interaction between group and bin was observed in the medial region [Fig. 4a, drug × bin: F (5, 50) = 4.47, *p* < 0.01]. In bins 1, 2, and 4, the number of CD68 microglia was higher in the IL-17A group than in the control group. No difference was observed between the groups in the distribution of CD68 microglia in the lateral region [Fig. 4b, effect of drugs: F (1, 50) = 0.84, N.S.]. Taken together, these results indicate that microglia accumulate in the ventricular zone and subventricular zone of IL-17A-treated mice, and that they are active microglia with upregulated phagocytic ability.

## Discussion

We previously reported that the activity and density of Iba1^+^ microglia was decreased in the dentate gyrus of the hippocampus by continuous upregulation of serum IL-17A using RORγt Tg mice (overexpression of RORγt [26, 27], Sasaki et al., *in submission*). It is possible that a persistently high level of IL-17A stimulates suppression of the immune system to reduce microglial activity. In this study, to directly evaluate the effect of IL-17A on microglia in vivo, we injected recombinant IL-17A into the lateral ventricle during the midgestational period and demonstrated that it increased the number of CD68^+^ microglia with high phagocytic ability at E18.5. This indicates that IL-17A administration activates microglia and enhances phagocytic activity, but no increase in their density in mice. The results are unusual, because activation of microglia is often accompanied by cell proliferation.

There are three paths for microglia to enter the brain during prenatal development: the pia mater, the ventricular membrane, and the choroid plexus [10]. Invaded microglia are observed on both the meningeal and ventricular side of the cortex. Among them, amoeboid-type microglia are frequently observed in the ventricular zone [28], and play a key role in the phagocytosis of neural progenitor cells and in regulation of the number of neural cells [12]. In this study, the number of CD68^+^ microglia in the ventricular and subventricular zones was higher in the IL-17A-treated group than in the saline group. This suggests that the microglia may excessively phagocytose neural progenitor cells in the ventricular zone after IL-17A administration. Another possibility is that IL-17A may have altered microglial movement during embryogenesis, namely migration from the ventricular surface into the cortex from the late fetal to the postnatal period [28, 29], and changed their morphology from the amoeboid to the ramified form. On this point, IL-17A-induced inflammation may trap microglia around the ventricular surface. It will be needed to understand the detailed molecular mechanisms (e.g., interaction with purinergic [30] or CX3CR1/CX3CL1 systems [31]) by which IL-17A alters microglial localization and activity.

The microglial accumulation, especially in the medial part of the cerebral cortex, including the cingulate cortex and the knee of the corpus callosum, was remarkable in the IL-17A-treated group. Interestingly, a previous study showed that intraventricular injection of IL-17A resulted in thinned cortical plates in the medial, but not lateral region of the brain [1]. CD68^+^ microglia in the medial region may phagocytose progenitor cells of excitatory neurons after IL-17A administration, which leading to disturbance of E/I balance in the cerebral cortex [32]. In mice, microglia accumulate around the corpus callosum, indusium griseum, medial zipper, and subcallosal sling around E17.5 and express CD68 [33]. Activated microglia that accumulate in the corpus callosum regulate guidance of callosal axons by their phagocytosis and release of neurotrophic factors [34]. IL-17A may have enhanced the tendency of microglia to cluster around the corpus callosum through their activation. It may lead to malformation of commissural fibers, such as those found in autistic people [35]. This study observed the effects of IL-17A administration on microglia at E18.5; however, the effects of IL-17A on changes in microglial dynamics and activity need to be tracked and examined over time.

## Supplementary information

**Additional file 1: Figure S1**. Representative photographs of Iba1 immunostaining after exposure to saline (upper panel) and IL-17A (lower panel). Blue: DAPI, Green: Iba1. Scale bars = 200 Μ*m. pia*: pia mater, Cx: cortex, LV: lateral ventricle, SVZ: subventricular zone, VZ: ventricular zone. L: lateral, M: medial.

**Additional file 2: Figure S2**. Magnified image of Iba1 immunostaining of E18.5 cortex exposed to saline. Arrowheads and arrows indicate round-form and ramified microglia, respectively. Blue: DAPI, Green: Iba1. Scale bars = 150 Μ*m. pia*: pia mater, Cx: cortex, LV: lateral ventricle, SVZ: subventricular zone, VZ: ventricular zone. L: lateral, M: medial.

**Additional file 3: Figure S3**. High-power magnified view of Iba1^+^ cells in E18.5 ventricular zone exposed to saline (upper panel) and IL-17A (lower panel). Note that microglia with few protrusions and large cell soma accumulate in the ventricular zone in the IL-17A administration group. Blue: DAPI, Green: Iba1. Scale bars = 20 μm. LV: lateral ventricle, VZ: ventricular zone. L: lateral, M: medial.

## Data Availability

The datasets, which were used and/or analyzed in the current study, are available from the corresponding author on reasonable request.

## Abbreviations

CC: Corpus callosum
Cx: Cortex
E: Embryonic day
Iba1: Ionized calcium binding adaptor molecule 1
IL: Interleukin
i.p.: intraperitoneal
L: Lateral
M: Medial
LV: Lateral ventricle
MIA: Maternal immune activation
Pia: Pia mater
PBS: Phosphate-buffered saline
poly(I:C): polyinosinic-polycytidylic acid
SC: Somatosensory cortex
SVZ: Subventricular zone
Th 17 cells: T helper 17 cells
VZ: Ventricular zone
