# Supplementary figures and images for: Intraventricular IL-17A administration activates microglia and alters their localization in the mouse embryo cerebral cortex
Source: Mol Brain. 2020 Jun 16;13:93. doi: 10.1186/s13041-020-00635-z (PMC7298827; doi:10.1186/s13041-020-00635-z)

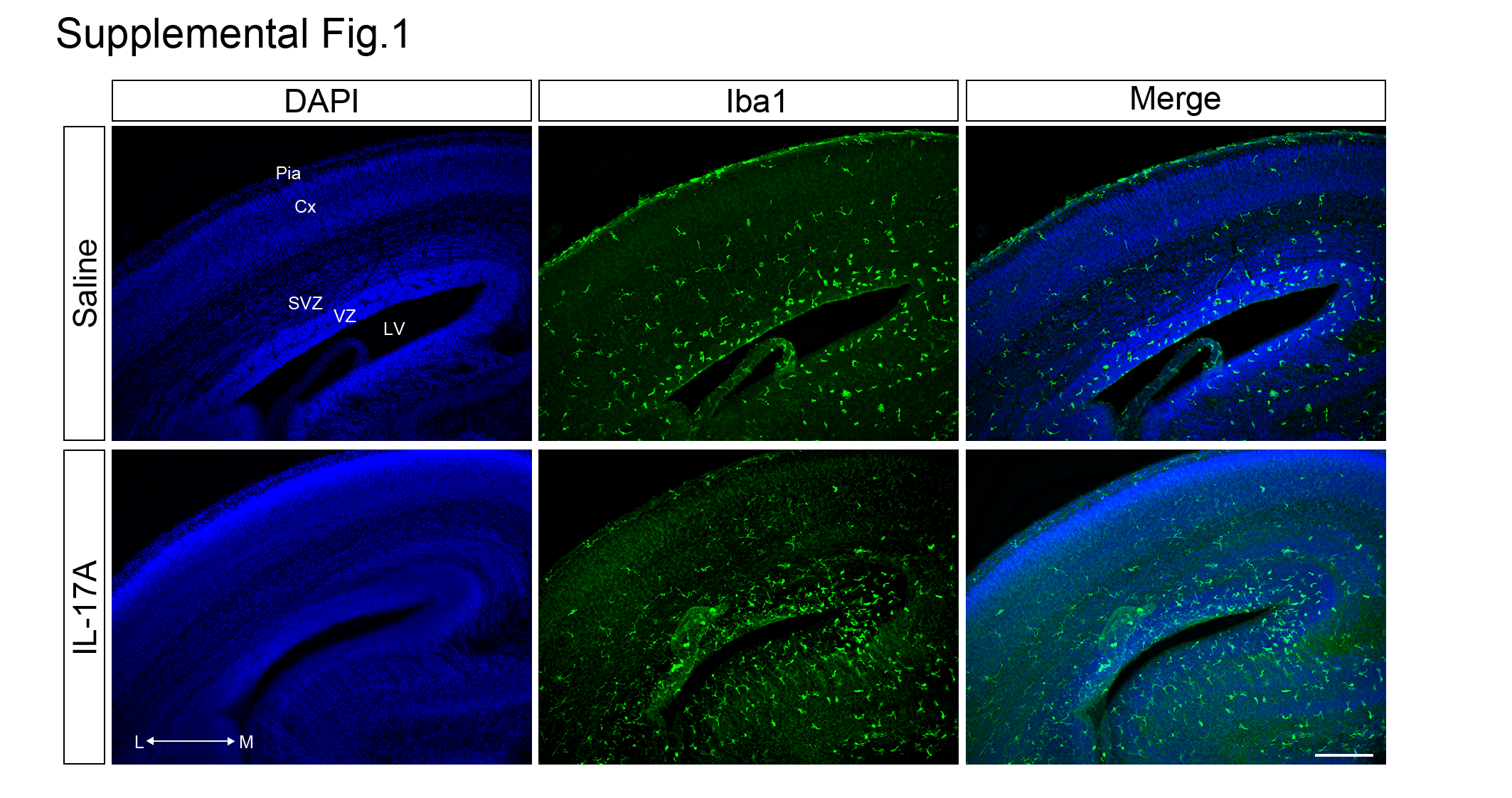

Supplement: Supplementary file 1 — Additional file 1: Figure S1. Representative photographs of Iba1 immunostaining after exposure to saline (upper panel) and IL-17A (lower panel). Blue: DAPI, Green: Iba1. Scale bars = 200 Μm. pia: pia mater, Cx: cortex, LV: lateral ventricle, SVZ: subventricular zone, VZ: ventricular zone. L: lateral, M: medial. [file 13041_2020_635_MOESM1_ESM.tif]

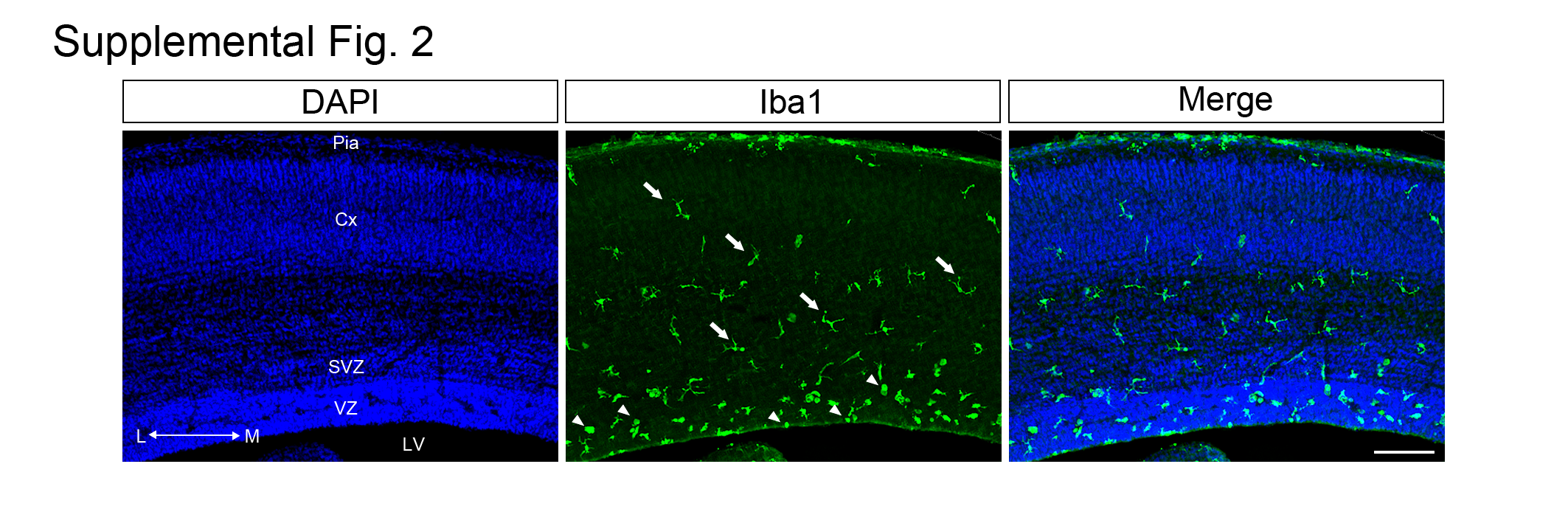

Supplement: Supplementary file 2 — Additional file 2: Figure S2. Magnified image of Iba1 immunostaining of E18.5 cortex exposed to saline. Arrowheads and arrows indicate round-form and ramified microglia, respectively. Blue: DAPI, Green: Iba1. Scale bars = 150 Μm. pia: pia mater, Cx: cortex, LV: lateral ventricle, SVZ: subventricular zone, VZ: ventricular zone. L: lateral, M: medial. [file 13041_2020_635_MOESM2_ESM.tif]

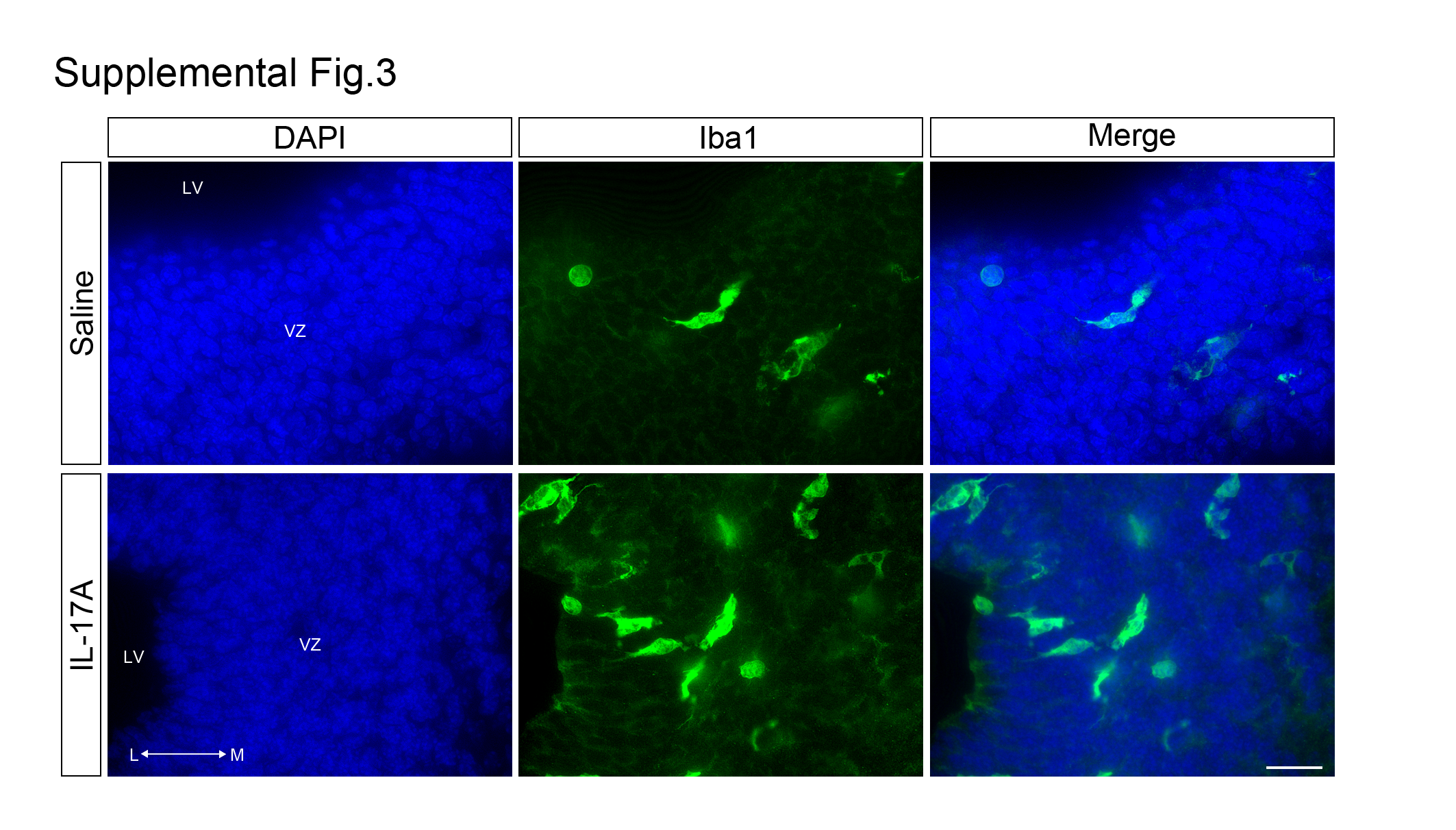

Supplement: Supplementary file 3 — Additional file 3: Figure S3. High-power magnified view of Iba1+ cells in E18.5 ventricular zone exposed to saline (upper panel) and IL-17A (lower panel). Note that microglia with few protrusions and large cell soma accumulate in the ventricular zone in the IL-17A administration group. Blue: DAPI, Green: Iba1. Scale bars = 20 μm. LV: lateral ventricle, VZ: ventricular zone. L: lateral, M: medial. [file 13041_2020_635_MOESM3_ESM.tif]
